# An fMRI study of facial emotion processing in children and adolescents with 22q11.2 deletion syndrome

**DOI:** 10.1186/1866-1955-7-1

**Published:** 2015-01-02

**Authors:** Rayna Azuma, Quinton Deeley, Linda E Campbell, Eileen M Daly, Vincent Giampietro, Michael J Brammer, Kieran C Murphy, Declan GM Murphy

**Affiliations:** School of International Liberal Studies, Waseda University, Tokyo, Japan; Department of Forensic and Neurodevelopmental Science, Institute of Psychiatry, King’s College London, London, UK; National Autism Unit, Bethlem Royal Hospital, SLAM NHS Foundation Trust, London, UK; School of Psychology, University of Newcastle, Newcastle, Australia; Department of Neuroimaging, Institute of Psychiatry, King’s College London, London, UK; Department of Psychiatry, Royal College of Surgeons in Ireland, Beaumont Hospital, Dublin, Ireland; Institute of Psychiatry, Sackler Institute for Translational Neurodevelopment, King’s College London, London, UK

**Keywords:** Velo-cardio-facial syndrome (VCFS), 22q11.2 deletion syndrome (22q11DS), Emotion, fMRI, Children, Social cognition

## Abstract

**Background:**

22q11.2 deletion syndrome (22q11DS, velo-cardio-facial syndrome [VCFS]) is a genetic disorder associated with interstitial deletions of chromosome 22q11.2. In addition to high rates of neuropsychiatric disorders, children with 22q11DS have impairments of face processing, as well as IQ-independent deficits in visuoperceptual function and social and abstract reasoning. These face-processing deficits may contribute to the social impairments of 22q11DS. However, their neurobiological basis is poorly understood.

**Methods:**

We used event-related functional magnetic resonance imaging (fMRI) to examine neural responses when children with 22q11DS (aged 9–17 years) and healthy controls (aged 8–17 years) incidentally processed neutral expressions and mild (50%) and intense (100%) expressions of fear and disgust. We included 28 right-handed children and adolescents: 14 with 22q11DS and 14 healthy (including nine siblings) controls.

**Results:**

Within groups, contrasts showed that individuals significantly activated ‘face responsive’ areas when viewing neutral faces, including fusiform-extrastriate cortices. Further, within both groups, there was a significant positive linear trend in activation of fusiform-extrastriate cortices and cerebellum to increasing intensities of fear. There were, however, also between-group differences. Children with 22q11DS generally showed reduced activity as compared to controls in brain regions involved in social cognition and emotion processing across emotion types and intensities, including fusiform-extrastriate cortices, anterior cingulate cortex (Brodmann area (BA) 24/32), and superomedial prefrontal cortices (BA 6). Also, an exploratory correlation analysis showed that within 22q11DS children reduced activation was associated with behavioural impairment—social difficulties (measured using the Total Difficulties Score from the Strengths and Difficulties Questionnaire [SDQ]) were significantly negatively correlated with brain activity during fear and disgust processing (respectively) in the left precentral gyrus (BA 4) and in the left fusiform gyrus (FG, BA 19), right lingual gyrus (BA 18), and bilateral cerebellum.

**Conclusions:**

Regions involved in face processing, including fusiform-extrastriate cortices, anterior cingulate gyri, and superomedial prefrontal cortices (BA 6), are activated by facial expressions of fearful, disgusted, and neutral expressions in children with 22q11DS but generally to a lesser degree than in controls. Hypoactivation in these regions may partly explain the social impairments of children with 22q11DS.

**Electronic supplementary material:**

The online version of this article (doi:10.1186/1866-1955-7-1) contains supplementary material, which is available to authorized users.

## Background

22q11.2 deletion syndrome (22q11DS), also known as velo-cardio-facial syndrome (VCFS), is a genetic disorder associated with a microdeletion in chromosome 22q11.2 [1–3]. It is the most common microdeletion syndrome with an estimated prevalence of 1 in every 4,000 live births [4–6]. While the physical phenotype is variable, commonly reported features include characteristic facial dysmorphology, congenital heart disease, and cleft palate [7, 8]. A characteristic behavioural phenotype in 22q11DS has also been described—with high rates of schizophrenia, attention deficit (hyperactivity) disorder (ADD, ADHD) [3, 8–13], autistic spectrum disorders, anxiety disorders, and emotional instability [12–17]. In addition, children and adults with 22q11DS typically have mild intellectual disabilities and a characteristic cognitive profile—with particular deficits in visual-perceptual function and social and abstract reasoning [3, 17–26]. It has been suggested by some that these cognitive deficits may contribute to the social impairments frequently observed in 22q11DS [17].

It is well established that children with 22q11DS have problems with social interaction, and perhaps, especially with peer relations rather than with adult figures [27–29]. For instance, it has been reported that people with 22q11DS typically show a ‘bland affect’ with minimal facial expression, in addition to disinhibited and impulsive or serious and shy extremes of behaviour [17, 25, 30, 31]. Furthermore, it has been reported that 20%–50% of children and adolescents in their sample of 22q11DS exhibited some ‘autism spectrum problem [13, 16, 25, 32–35].’ While reported prevalence of autistic spectrum disorder (ASD) among 22q11DS varies widely from study to study, it is nevertheless considerably higher than in the general population which is around 1% to 1.5% [36–43]. Hence, impairment in social function may be a central feature of 22q11DS [44]. A recent study has reported that weaker functional connectivity between the posterior cingulate gyrus and other default mode network nodes (such as the precuneus, precentral gyrus, and left frontal pole) observed in individuals with 22q11DS was correlated with lower social competence [44]. However, cognitive and/or functional neural substrates underlying social impairment are undetermined.

The ability to perceive and respond to facial expressions is crucial to managing social interactions and relationships, and deficits in face processing have often been reported in clinical populations with social interaction problems [45, 46]. For instance, abnormalities in the expression and recognition of emotions have been reported in people with ASD [47–49]. In 22q11DS, deficits in memory for faces have been reported [18, 50], and recent behavioural studies have shown that children and adolescents with 22q11DS have difficulties identifying faces and facial emotions [18, 51–56]. For instance, during face identification and emotion recognition tasks, children and adolescents with 22q11DS (as compared to controls) make more errors and pay preferential attention to the mouth rather than the eyes [51, 52]. These deficits in the recognition of faces and facial emotion may reflect inefficient strategies employed by the individuals with 22q11DS and/or biological differences during facial emotion processing.

In healthy humans, face processing engages a variety of brain regions, some of which are common to all emotion types (e.g. core visual analysis areas of striate and extrastriate cortex, particularly the fusiform gyrus [FG]) [57, 58]. Core visual analysis areas project to ‘downstream’ components of face responsive systems, in which cortical and subcortical regions are differentially activated depending on stimulus features (e.g. neutral or fearful face) and cognitive task (e.g. emotion recognition) [59, 60]. Further, identification of the emotional valence of faces (and other stimuli) and the generation of emotional responses typically require activity in a predominantly ventral neural system. This includes the amygdala, insula, ventral striatum, and ventral regions of the anterior cingulate cortex and prefrontal cortex [46, 57]. Furthermore, within healthy populations, across different emotions, as emotion intensity of a facial expression increases, there is differential engagement of components of face responsive networks such as core visual analysis areas (including the FG) and limbic regions [60]. For example, the amygdala and visual cortices are co-activated in response to fearful faces. It has been proposed that visual cortex activation is boosted by ‘feedback’ influences from the amygdala [61, 62] and that this may contribute to the detection and memorization of important social cues [63]. Another structure that is implicated in the processing of emotion is the cerebellum [64–67]. Studies have implicated the cerebellum in various aspects of emotional processing such as identification of emotion in a speaker’s voice [68], induction of positive and negative emotion [69, 70], processing of positive and negative emotional stimuli [71], and fear conditioning [72, 73]. It has been suggested that cerebellar activation may be associated with processing of ‘primitive’ emotion [74], but the exact nature of the contribution of the cerebellum to emotion processing is still unclear.

To date, two imaging studies have investigated facial emotion processing in 22q11DS. In the first functional magnetic resonance imaging (fMRI) study [75], eight adults with 22q11DS and nine age- and IQ-matched controls were scanned during an incidental (gender discrimination) task. Two types of facial emotions (happy or angry) and neutral faces were presented in a block design. Individuals with 22q11DS showed significant hypoactivation of the right insula and of the frontal regions and higher activation of the occipital regions compared to the controls. It was suggested that these findings may be partially explained by dysmaturation of white matter tracts in 22q11DS [76, 77] and may underlie social-emotional dysfunction seen in this population. However, this study employed a block design that mixed both ‘happy’ and ‘angry’ faces—making it impossible to distinguish neural responses to two very different emotions (i.e. one positive and one negative) or neutral faces.

A more recent event-related fMRI study examined brain activity in response to fearful and neutral facial expressions in young people with 22q11DS and controls during an incidental (faces/houses categorization) task [78]. The authors reported a lack of face-selective activation in FG and a lack of modulation of the amygdala and superior temporal sulcus by fearful expressions. These findings were associated with the absence of repetition-suppression effect in 22q11DS, both of which were present in the controls. The authors suggested that these functional abnormalities in the FG, amygdala, and other face-related areas may underlie social deficits in 22q11DS [78]. However, this study only employed one emotion type (fear) at a single intensity (prototypic), whereas normal social interaction involves different types of facial expressions at different intensities [60]. Further, one third of the 22q11DS group had psychotic symptoms, which may have contributed to the group differences observed in their study. For example, several studies have reported abnormal brain activity during facial emotion processing in people with psychotic illness [79–81]; similarly, a recent study of facial emotion processing in 22q11DS reported differences in brain activations between 22q11DS volunteers with psychotic symptoms and those without [78].

In summary, previous studies point to abnormalities in the neural responses associated with processing facial expressions in 22q11DS [75, 78]. However, it is unclear whether (and how) the functional anatomy of these neural responses may differ according to different types of primary emotions and across a range of intensities. In order to address these issues, studies are required in young people without psychotic symptoms and across more than one emotion type and intensity.

We therefore employed a cross-sectional event-related design to investigate neural responses to neutral faces and to two facial emotions (fear, disgust) with high (100%) and mild (50%) emotional intensities in children and adolescents with 22q11DS. We selected fear and disgust out of the basic human emotional expressions because of their significance in the development of socialization skills. For instance, it has been suggested that individuals learn to avoid behaviours that are associated with fearful expressions as they elicit an aversive arousal response [82]. We also included disgust as it signals socially unacceptable behaviours as well as aversive stimuli (e.g. odours and/or tastes associated with rotten food) [83]. Based on previous studies, we predicted that typically developing children would show differential activation of the limbic area depending on emotion type (e.g. greater amygdala activation for fear and insula activation for disgust) [45, 46]. Given the evidence of reduced recognition of facial expressions of emotion in children with 22q11DS, we tested the main hypothesis that they would show increased limbic and visual cortical responses as emotion intensity is increased but that these responses would be significantly less than those of healthy controls for both emotion types and intensities. We also conducted exploratory correlation analysis, within the 22q11DS group, of measures of social behaviour, as measured by the Strengths and Difficulties Questionnaire (SDQ) [84–86] and neural activity in order to determine what, if any, functional abnormalities may be associated with social difficulties in people with 22q11DS.

## Methods

### Participants

We studied 28 children and adolescents; 14 with 22q11DS and 14 normally developing controls. The 22q11DS group comprised seven males and seven females, aged 9–17 years (mean age ± standard deviation, 13 ± 2 years), and the healthy controls included nine males and five females, aged 8–17 years (13 ± 3 years) (see Table 1 for demographic data). Where possible, we used sibling controls in order to match for socio-cultural background. Nine of the control group were siblings, and five were recruited from the local community. All were right-handed.

**Table 1:** **Demographic data and task performance**

	22q11DS	Control	***p*** value
	***n*** = 14	***n*** = 14 (9)	
Age range (mean ± SD)	9–17 (13 ± 2)	8–17 (13 ± 3)	0.942
Sex: M/F	7/7	9(5)/5(4)	
FSIQ (WISC-III)	66 ± 7	114 ± 16	0.000^**^
PIQ (WISC-III)	67 ± 8	108 ± 19	0.000^**^
VIQ (WISC-III)	70 ± 12	111 ± 16	0.000^**^
ASQ	*n* = 12	*n* = 8	
	6 ± 4	2 ± 3	0.048^*^
ADHD index	*n* = 11	*n* = 9	
	60 ± 10	48 ± 5	0.039^*^
SDQ	*n* = 11	*n* = 9	
Total difficulties	13.9 ± 7.1	6.2 ± 4.1	0.011^*^
Emotional symptoms	3.6 ± 3.3	0.7 ± 0.7	0.117^*^
Conduct problems	2.1 ± 1.6	1.6 ± 1.6	0.471
Hyperactivity	5.2 ± 3.3	3.2 ± 2.9	0.182
Peer problems	3 ± 2.7	0.8 ± 1.4	0.040^*^
Prosocial behaviour^a^	7.9 ± 1.6	8.6 ± 1.4	0.354
Gender discrimination task			
Fear accuracy	73 ± 20	83 ± 17	0.15
Fear response time	771 ± 212	842 ± 109	0.279
Disgust accuracy	81 ± 9	83 ± 17	0.606
Disgust response time	883 ± 139	840 ± 126	0.408

Accuracy and response time for the gender discrimination task for 22q11DS group and the controls. ADHD index which is a subscale of the ‘Conners Rating Scales’ (CRS).

*FSIQ* full scale IQ, *PIQ* performance IQ, *VIQ* verbal IQ, *ASQ* Autism Screening Questionnaire, *SDQ* Strengths and Difficulties Questionnaire.

^*^Significant at a trend level *p* < 0.05; ^**^significant at *p* < 0.001; ^a^reverse scale.

All participants underwent psychiatric and physical examination and routine blood tests. We excluded participants with a clinically detectable co-morbid psychiatric disorder or physical disorder affecting brain function (e.g. ADHD, hypothyroidism, or epilepsy) or with a clinically abnormal MRI scan—as determined by a neuroradiologist. None of the participants had any psychotic symptoms, and all were drug free at the time of testing.

The 22q11DS volunteers were recruited through the 22q11 (UK) Support group and the Behavioural Genetics Clinic at the Maudsley Hospital/Institute of Psychiatry, King’s College London. 22q11DS was diagnosed by fluorescence *in situ* hybridization (FISH) using the N25 probe (Oncor Inc.). As exclusion criteria, we recruited only those who had been able to successfully complete MRI scanning for another study [87] and who had a minimum Full Scale IQ (FSIQ) of 55, as the FSIQ of children with 22q11DS tends to range from normal to moderately learning disabled with a mean FSIQ of about 70 [17, 21]. The ‘Autism Screening Questionnaire’ (ASQ) was administered in order to measure autistic traits with a cut-off score of 14 for those who may have autism and who should have a more complete assessment [88]. The ASQ score was significantly higher in the 22q11DS group than in the controls (*t*(18) = 2.12, *p* < .05), but none scored above the cut-off. We measured ADHD symptoms with the ‘Conners Rating Scales’ (CRS) [89, 90]. Among those who agreed to provide the data, children with 22q11DS scored significantly higher (60 ± 10) than the controls (48 ± 5). Further, in view of the high rates of schizotypy reported in 22q11DS adults [9], an assessment of schizotypal traits was also performed. However, since there was no validated measure for schizotypy in learning-disabled children, we constructed a preliminary comparative schizotypy scale derived from DSM-IV [91]. Of those who agreed to provide the information, 6 out of 11 children with 22q11DS scored at least one positive rating (score range 0–7, mean ± SD: 2 ± 2), whereas none of the 9 controls did. This may indicate a higher level of schizotypy traits in the VCFS sample, although the schizotypy scale itself needs to be validated. The Wechsler Intelligence Scale for Children-III (WISC-III) [92] was used to assess general intellectual function. The mean FSIQ and standard deviation for individuals with 22q11DS and controls were 66 ± 7 and 114 ± 16, respectively. All the participants and/or their parents gave written informed consent/assent as approved by the local research ethics committee (Institute of Psychiatry, South London and Maudsley Trust, 067/00).

The parent’s version of the SDQ [84–86, 93] was used to measure emotional symptoms, conduct problems, hyperactivity, peer problems, and prosocial behaviour. The Total Difficulties Score is generated by summing the scores from all the scales except the Prosocial scale (where normal-abnormal direction of the scores is the opposite from the others). The SDQ is a well-validated behavioural screening questionnaire and the Total Difficulties Score provides an aggregate measure of difficulties in the behaviours, emotions, and relationships of children and young people.

### Functional neuroimaging task

Each volunteer participated in two 6-min event-related fMRI experiments. In each experiment, participants were presented with facial expressions of one of two primary emotions (disgust, fear) and neutral expressions from a standardized series of prototypical facial expressions posed by ten different volunteers [60, 94]. The ten prototypical expressions of primary emotions were further manipulated by morphing software to depict expressions of mild (50%) and high (100%) intensity along the neutral-prototypical expression continuum [94]. Thus, there were ten faces with three levels of intensity, each of which was presented twice to give a total of 60 stimuli per experiment.

For example, in the ‘disgust’ experiment, participants viewed prototypically disgusted (i.e. expressions of 100% disgust), mildly disgusted (i.e. expressions of 50% disgust), and neutral expressions. Each facial stimulus was presented for 2 s. All stimuli were presented in a pseudorandomized order to avoid successive presentation of expressions of the same emotional intensity. Each stimulus type was preceded by similar numbers of each of the other two stimulus types to minimize the effect of the preceding stimulus type on neural responses to the stimulus of interest. The duration of the interstimulus interval (ISI) varied from 3 to 8 s (average 4.9 s) according to a Poisson distribution to prevent participants predicting the timing of the next stimulus presentation. During the ISI, participants viewed a fixation cross (see Figure one, Surguladze et al., for the design [60]). In subsequent analyses, the fixation cross was used as the baseline stimulus in each of the experiments. Participants were requested to decide on the sex of each face and press one of two buttons accordingly with the right thumb. The participants were familiarized with the stimuli and task procedures before scanning. In prescan testing where participants were shown the same faces as the test version but with neutral expressions only, they were all able to identify the sex of the faces correctly.

### Image acquisition

Magnetic resonance (MR) images were acquired using a GE Signa 1.5 Tesla system (General Electric, Milwaukee, WI) with an operating console and software (Advanced Nuclear Magnetic Resonance, Woburn, MA) for gradient echo echoplanar imaging (EPI) at the Maudsley Hospital, London, UK. A quadrature birdcage headcoil was used for RF transmission and reception. An inversion recovery EPI dataset was acquired at 43 near-axial 3-mm-thick planes parallel to the AC-PC line: TE 73 ms, TI 180 ms, TR 16 s, in-plane voxel size 1.72 × 1.72 mm, interslice gap 0.3 mm, and matrix size: 128 × 128 pixels. This higher-resolution EPI dataset provided whole brain coverage and was later used to register the fMRI datasets acquired from each individual in standard stereotactic space. T2*-weighted images (180) depicting blood oxygenation level-dependent (BOLD) contrast were acquired at each of 16 near-axial non-contiguous 7-mm-thick planes parallel to the intercommissural (AC-PC) line: TE 40 ms, TR 2 s, in-plane voxel size 3.44 × 3.44 mm, interslice gap 0.7 mm, and matrix size: 64 × 64 pixels.

Visual stimuli were presented via a conventional PC and LCD projector system to a screen placed at the feet of the participant and projected to them via a well-positioned mirror, and overt responses were made using an MR-compatible 2-button box placed in the participant’s dominant right hand. Presentation of all stimuli and recording of all participant responses were synchronized to the imaging system. The total functional image acquisition time for each experiment was 6 min.

### Neuroimaging data analysis

#### Individual brain activation maps

Data were analysed using a non-parametric approach with XBAM (version 4) brain image analysis software developed at the Institute of Psychiatry [95]. The images were first processed to minimize motion-related artefacts [96]. A 3D volume consisting of the average intensity at each voxel over the whole experiment was calculated and used as a template. The 3D image volume at each time point was then realigned to this template. Following realignment, data were detrended and smoothed using a Gaussian filter (FWHM 7.2 mm) to improve the signal-to-noise characteristics of the images. We did not exclude any subjects based on motion because mean motion in each of the *X*-*Y*-*Z* direction was under 1 mm for all of these subjects that were tested. This low level of motion artefact may be because we recruited 22q11DS and sibling participants from a sample that had successfully completed MRI structural scanning prior to this study.

Experimental responses were analysed by convolving each contrast of interest (neural responses to neutral versus baseline, emotional expressions versus baseline, respectively, and emotional expressions versus neutral) with two gamma variate functions (peak responses at 4 and 8 s, respectively). These two functions were chosen to encompass the known range of times to peak response following stimulus onset for BOLD effects. The best fit between the weighted sum of these convolutions and the time series at each voxel was computed using Friman’s constrained BOLD effect model [97]. Following the computation of the model fit, a goodness-of-fit statistic was computed. This consisted of the ratio of the sum of squares of deviations from the mean image intensity (over the whole time series) due to the model to the sum of squares of deviations due to the residual sum of squares (SSQ) ratio. Following the computation of the observed SSQ ratio at each voxel, the data were permuted by the wavelet-based method described in [98]. This allowed the data-driven calculation of the null distribution of SSQ ratios under the assumption of a no experimentally determined response. Using this distribution, it is possible to calculate the critical value of the SSQ ratio needed to threshold the maps at any desired type I error rate. The detection of activated voxels was then extended from the voxel-to-cluster level using the method described in detail by [99].

#### Group brain activation maps

The observed and permuted SSQ ratio maps for each individual were transformed into standard space [100] using a two-stage warping procedure described in detail in [95]. Group activation maps were then computed by determining the median SSQ ratio at each voxel (over all individuals) in the observed and permuted data maps (medians are used to minimize outlier effects). In the two-level clustering procedure (described in detail in Bullmore et al, 1999 [99]), the first (voxel-wise) thresholding was carried out at an uncorrected *p* value of 0.05 to give the maximum allowable sensitivity. In order to eliminate the resulting false positive activations, a second cluster-level thresholding step was carried out and the threshold of this second step was adjusted to give an expectation of less than one false positive cluster over the whole brain. Thus, the computation of a standardized measure of SSQ ratio at the individual level, followed by analysis of the median SSQ ratio maps over all individuals, treats intra- and interparticipant variations in effect separately. This constitutes a mixed-effect approach that allows for inferences from these results to be made about the larger population—in other words, people with 22q11DS as a whole (assuming a representative sample).

#### Within-group linear trend analysis

Voxel- and cluster-wise within-group differences in BOLD signal change to the two different types of facial expression versus the baseline were examined using repeated measures analysis of variance (ANOVA), with intensity (neutral, 50% intensity, 100% intensity) as the within-participant variable. Each ANOVA was constrained to detect brain regions demonstrating linear trends of activation—in other words, areas where activity changes in a stepwise way across levels of emotion intensity regardless of direction (i.e. both positive and negative trends).

#### Between-group and group-by-intensity interaction analysis

A 3 × 2 repeated measures ANOVA was undertaken to determine voxel- and cluster-wise between-group differences in BOLD signal to the two different types of facial expression versus baseline, with intensity (neutral, 50% intensity, 100% intensity) as the within-participant variable for each emotion type and group (22q11DS, controls) as the between-group variable. Group × intensity interactions refer to brain regions showing differences in the effect of changes in emotion intensity on neural response in children with 22q11DS relative to healthy controls. The main effects of group refer to the differences between children with 22q11DS relative to controls in the neural response to all emotion intensities (neutral, mild, and intense) considered together.

#### Post hoc analysis

Further tests of between-group differences in neural response to each of the three different emotion expression-baseline contrasts for each separate emotion were examined with *post hoc* ANOVAs. In addition, neural responses to neutral expressions from both experiments were combined to increase experimental power.

#### Correlations between brain activity during facial emotion processing and social behaviour

We also conducted an exploratory correlation analysis, within the 22q11DS group, of measures of social behaviour and brain activity in regions where healthy controls activated significantly more than the 22q11DS group (i.e. employing a ‘mask’ derived from brain regions showing a main effect of group on neural responses to facial expressions for fearful and disgusted expressions respectively.) Since not all the guardians agreed to complete these questionnaires, we could only include data from 11 of the 14 children with 22q11DS for this analysis. The aim was to determine what, if any, functional abnormalities might be associated with social difficulties in people with 22q11DS as measured by SDQ total difficulties score (abnormal score ≥17).

## Results

### Demographic details and behavioural results

There were no significant between-group differences in age (*t*(26) = .07, *p* > .05), response accuracy (Fear: *t*(26) = 1.48, *p* > .05; Disgust: *t*(26) = .52, *p* > .05), or reaction times (Fear: *t*(26) = 1.12, *p* > .05; Disgust: *t*(26) = .84, *p* > .05). However, as noted above, as expected, the healthy controls had a significantly higher FSIQ (*t*(26) = 10.4, *p* < .001) (see Table 1).

### Face versus fixation cross contrasts

Results of contrasts of neutral faces versus fixation cross, and prototypic (100% intensity) facial emotions versus fixation cross for each group, are in the Additional file 1. In summary, these contrasts revealed fusiform and extrastriate activations in both children with 22q11DS and controls, along with activation in other brain regions involved in face perception (e.g. superior temporal gyri, insula, anterior cingulate gyri, and medial frontal gyri).

### Within group linear trend analysis

Table 2 shows results of repeated measures ANOVA of neural responses to facial expressions of neutral, mild, and intense emotion for fearful and disgusted expressions, respectively. Positive linear trends (i.e. increased levels of activation as the intensity of the facial expression increased) were found for fearful expressions in both groups and for disgusted expressions in the 22q11DS group (Table 2). Negative linear trends of activation (i.e. decreased levels of activation as the intensity of the facial expression increased) to disgusted expressions were present in the controls (Table 2). In summary, we found significant positive trends of activation in fusiform-extrastriate regions and cerebellum to increasing intensities of fear in both groups, and positive trends in similar areas to increasing intensities of facial disgust in children and adolescents with 22q11DS.

**Table 2:** **The effect of emotional intensity**

		22q11DS					Controls				
Brain region	BA	X	Y	Z	Size	***p*** value	X	Y	Z	Size	***p*** value
FEAR											
Middle occipital gyrus											
Right	18	29	-78	-7	8						
Lingual gyrus											
Right	18	18	-78	-7	5						
Precentral gyrus											
Left	4						-36	-22	48	76	0.007657
Postcentral gyrus											
Left	2						-47	-19	31	10	
Inferior parietal lobule											
Left	40						-40	-30	42	16	
Cerebellum											
Left							-40	-63	-29	83	0.006032
Right		29	-78	-13	62	0.00486	32	-63	-29	83	0.007889
DISGUST											
Inf-post temporal lobe											
Right	37	36	-56	-2	96	0.008705					
Middle occipital gyrus											
Right	18	32	-78	4	17						
Inferior occipital gyrus											
Right	19	32	-78	9	7						
Fusiform gyrus											
Right	37	36	-56	-7	37						
Middle temporal gyrus											
Right	21	54	-37	-2	6						
Lingual gyrus											
Right	18						*14*	-*85*	-*7*	*106*	*0.004004*
Medial frontal gyrus											
Left	6						-*4*	*4*	*48*	*66*	*0.005116*
Cingulate gyrus											
Left	24						-*4*	*4*	*42*	*15*	
Cerebellum											
Right		29	-63	-13	9		*25*	-*70*	-*13*	*9*	

Brain regions showing significant trends of activation to increasing intensity of emotion (neutral, mild, intense) for fearful and disgusted expressions. Both groups showed positive trend (i.e. increasing activation with the increasing emotional intensity) for ‘fear’. For ‘disgust’, the 22q11DS group showed positive trend whereas the controls showed negative trends (i.e. decreasing activation with increasing emotional intensity—shown in *italics*). Statistical thresholds adjusted so as to get less than one false positive cluster per map.

### Between-group and group-by-intensity interaction analysis

**Figure 1:**
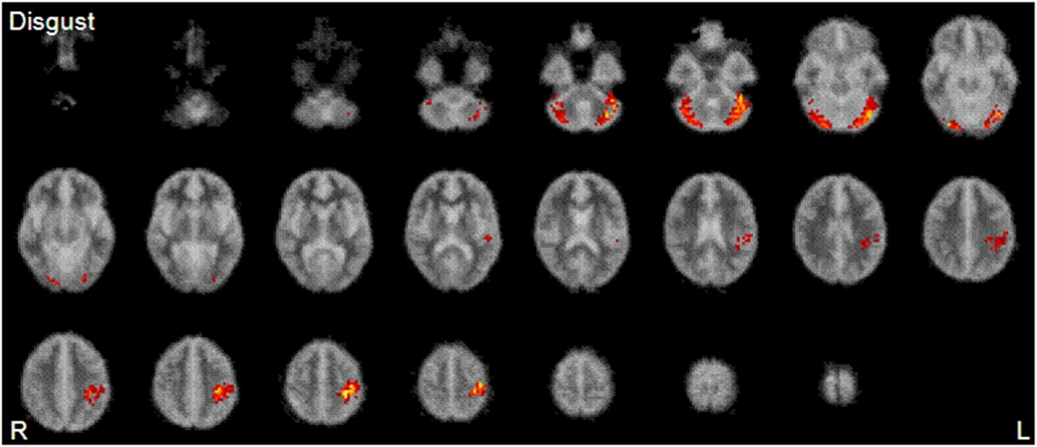
**Main effect of group analyses demonstrating regions where controls activate more than people with 22q11DS in response to disgusted expression.** Controls showed significantly greater activation in extrastriate and fusiform cortices than children with 22q11DS. Twenty-five 5.5-mm slices extend from *z* = -51.15 to *z* = 69.85. See Table 3 for a full description of the functional anatomy and peak Talairach coordinates.

**Figure 2:**
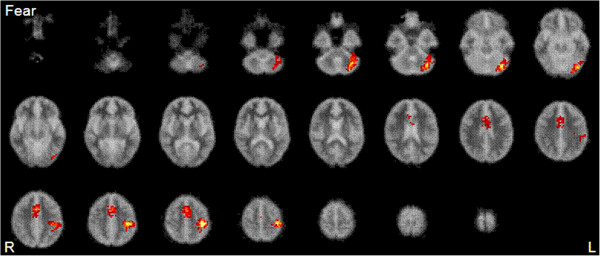
**Main effect of group analyses demonstrating regions where controls activate more than people with 22q11DS in response to fearful expression.** Controls showed significantly greater activation in extrastriate and fusiform cortices than children with 22q11DS. Twenty-five 5.5-mm slices extend from *z* = -51.15 to *z* = 69.85. See Table 3 for a full description of the functional anatomy and peak Talairach coordinates.

The main effects of group on neural response to expression (neutral, mild, and intense emotion) and interactions between group (controls, 22q11DS) and expression intensity (neutral, mild, and intense emotion) for fearful and disgusted expressions, respectively, are shown in Table 3. Figures 1 and 2 show brain activation maps of the main effects of group. Briefly, we found that, compared to healthy controls, children with 22q11DS showed reduced activations of the extrastriate cortices (including FG), postcentral gyrus, precentral gyrus, and cerebellum in response to the expressions of fear and disgust (i.e. main effects of group). Tests of interactions showed that there were significant group × intensity interactions in the left precentral gyrus for fear, whereas the significant interactions for disgust were found in the left precentral gyrus, the right cingulate gyrus, and the left cerebellum. This result indicates that neural ‘hyporesponsiveness’ in children and adolescents with 22q11DS varies depending on emotion type. Furthermore, the pattern of the results remained unchanged even when we repeated the analyses using FSIQ as a covariate to see if any differences observed might reflect the differences in FSIQ between the groups.

**Table 3:** **The main effect of group and group × intensity interaction**

		Fear					Disgust				
Brain region	BA	X	Y	Z	Size	***p*** value	X	Y	Z	Size	***p*** value
Controls > 22q11DS											
Fusiform gyrus											
Left	19	-36	-74	-13	27		-40	-70	-18	49	
Right	19						*29*	*-81*	*-13*	*119*	*0.003945*
Inferior occipital gyrus											
Left	19	-36	-78	-7	5						
Insula											
Left	13						-29	-30	26	10	
Superior frontal gyrus											
Right	6	*4*	*11*	*48*	*164*	*0.003543*					
Postcentral gyrus											
Left	3	-32	-30	48	37		*-29*	*-30*	*48*	*180*	*0.001517*
Precentral gyrus											
Left	4	*-36*	*-19*	*48*	*136*	*0.000273*	-36	-19	53	21	
Transverse temporal gyrus											
Left	41						-47	-22	9	**5**	
Cingulate gyrus											
Right	32	0	11	37	33						
Cerebellum											
Left		*-36*	*-79*	*-18*	*186*	*0.000273*	*-40*	*-44*	*-24*	*173*	*0.000305*
Right							25	-74	-24	40	
GROUP × INTENSITY											
Fusiform gyrus											
Left	19						-32	-74	-18	22	
Precentral gyrus											
Left	4	*-32*	*-22*	*53*	*50*	*0.005263*	*-29*	*-22*	*53*	*92*	*0.00246*
Medial frontal gyrus											
Right	6						0	-11	53	7	
Superior frontal gyrus											
Right	6						0	4	48	23	
Cingulate gyrus											
Right	24						*0*	*4*	*42*	*63*	*0.006812*
Postcentral gyrus											
Left	3	-32	-33	48	21		-32	-22	42	27	
Inferior parietal lobule											
Left	40						-47	-30	31	5	
Cerebellum											
Left							*-40*	*-70*	*-24*	*99*	*0.002271*

Brain regions showing main effects of group and group × intensity level interactions for each emotion experiment. Talairach coordinates in *italic print* indicate the most activated voxel within a cluster. Other Talairach coordinates represent other active areas in clusters (derived from decomposition of each cluster into contiguous slices, 5.72-mm diameter in the *z* dimension). Statistical thresholds adjusted so as to get less than one false positive cluster per map.

#### Post hoc analysis

To further clarify these group × intensity interactions and the main effects of group, separate *post hoc* two-way ANOVA comparisons of neutral, mild, and intense emotion for the fear and disgust experiments are shown in Tables 4 and 5, respectively. In brief, controls showed significantly higher activations than people with 22q11DS in response to fearful emotion in the postcentral gyrus, the left superomedial frontal gyrus, and the bilateral cerebellum across emotional intensities. For disgusted facial emotion, controls activated significantly more than the 22q11DS group in the left fusiform gyrus, left postcentral gyrus, and the bilateral cerebellum. We found that there were no brain regions where people with 22q11DS activated more than controls in response to fearful, disgusted, or neutral facial expressions.

**Table 4:** ***Post hoc*** **analyses on group × intensity interaction for fear**

		Intense				Mild				Neutral			
Brain region	BA	X	Y	Z	Size	X	Y	Z	Size	X	Y	Z	Size
Control > 22q11DS													
Fusiform gyrus													
Left	19					-29	-81	-13	24	-36	-70	-13	26
Right	19									*33*	*-78*	*-13*	*64*
Thalamus													
Left										-7	-15	15	7
Paracentral lobule													
Left	31									0	-11	48	13
Precentral gyrus													
Left	4	-32	-22	53	23	-36	-22	53	22				
Postcentral gyrus													
Left	3	*-33*	*-22*	*48*	*135*	*-33*	*-22*	*48*	*174*	*-40*	*-26*	*48*	*80*
Medial frontal gyrus													
Left	32	7	11	42	23	*0*	*11*	*42*	*128*				
Superior frontal gyrus													
Left	6	*7*	*11*	*48*	*99*								
Right	6					4	11	48	31				
Cingulate gyrus													
Left	24	-7	7	26	7					*0*	*15*	*37*	*95*
Right	32	11	11	37	22	11	11	37	27	0	-4	26	16
Cerebellum													
Left		*-33*	*-70*	*-24*	*135*	*-33*	*-67*	*-29*	*120*	*-36*	*-70*	*-18*	*163*
Right		*36*	*-70*	*-24*	*84*								

*Post hoc* between-group two-way ANOVA comparisons of neural responses to intense, mild, and neutral expressions, respectively, for fearful emotion experiment. Talairach coordinates in *italic print* indicate the most activated voxel within a 3D cluster. Other Talairach coordinates represent other active areas in clusters (derived from decomposition of each cluster into contiguous slices, 5.72-mm diameter in the *z* dimension). Statistical thresholds adjusted so as to get less than one false positive cluster per map.

**Table 5:** ***Post hoc*** **analyses on group × intensity interaction for disgust**

		Intense				Mild				Neutral			
Brain region	BA	X	Y	Z	Size	X	Y	Z	Size	X	Y	Z	Size
Control > 22q11DS													
Fusiform gyrus													
Left	19	-40	-70	-13	24					-36	-67	-13	23
Right										32	-78	-13	19
Inferior parietal lobule													
Left	40					-40	-37	37	32				
Insula													
Left	13	-29	-30	26	8					-43	-30	20	8
Precentral gyrus													
Left	4	-36	-19	53	6	-29	-26	53	18	-36	-15	53	14
Postcentral gyrus													
Left	3	*-29*	*-30*	*48*	*72*	*-29*	*-30*	*48*	*162*	*-33*	*-22*	*42*	*125*
Middle occipital gyrus													
Left	18	-22	-85	-7	5								
Medial frontal gyrus													
Left	32					*0*	*7*	*42*	*72*				
Cingulate gyrus													
Right	24					7	7	37	19				
Cerebellum													
Left		*36*	*-48*	*-24*	*78*	*-40*	*-70*	*-18*	*85*	*-36*	*-67*	*-18*	*173*
Right		*-43*	*-59*	*-29*	*148*	*25*	*-81*	*-24*	*78*	*33*	*-78*	*-18*	*137*

*Post hoc* between-group two-way ANOVA comparisons of neural responses to intense, mild, and neutral expressions, respectively, for disgusted emotion experiment. Talairach coordinates in *italic print* indicate the most activated voxel within a cluster. Other Talairach coordinates represent other active areas in clusters (derived from decomposition of each cluster into contiguous slices, 5.72-mm diameter in the *z* dimension). Statistical thresholds adjusted so as to get less than one false positive cluster per map.

### Correlation with behavioural measures

**Figure 3:**
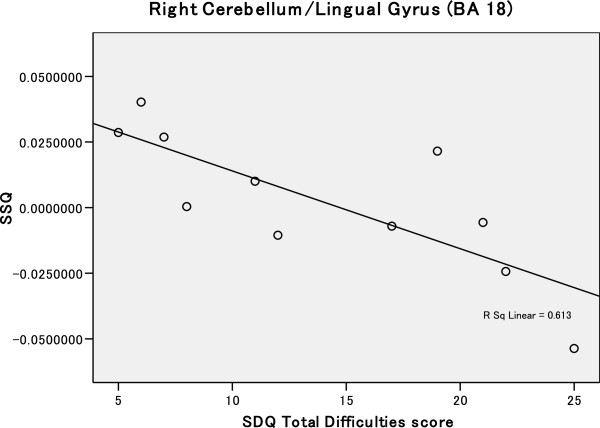
**Correlation between SDQ total difficulties score and brain activity (SSQ) in brain regions that show reduced activation to disgusted expressions compared to healthy controls.** A significant negative correlation between SDQ total difficulties score and brain activity (SSQ) was demonstrated within the right cerebellum/lingual gyrus (BA 18). Statistical thresholds adjusted so as to get less than one false positive cluster per map.

Eleven children with 22q11DS and nine siblings provided the parent’s version of the SDQ. The siblings scored well under the cut-off scores for all of the subscales, while children with 22q11DS scored in the borderline—abnormal range for the ‘Emotional Symptoms’ and ‘Peer Problems’ scores which are related to social behaviour (see Table 1). These two subscales were the only ones that yielded significant group difference. We used the ‘Total Difficulties Score’ for exploratory correlation analysis which is generated by summing the subscale scores including these two because it is a well-established measure for screening for behavioural, emotional, and social difficulties among children both at home and school [84, 93, 101, 102]. In children with 22q11DS, we found a significant correlation between decreasing brain activations as the SDQ Total Difficulties Score increased, to fearful expressions in the left precentral gyrus (BA 4, *r* = 0.453), and to disgusted expressions in the left fusiform gyrus (BA 19, *r* = 0.447), right lingual gyrus (BA 18) (Figure 3), and bilateral cerebellum (*r* = 0.613).

## Discussion

We carried out a cross-sectional event-related fMRI study in children and adolescents with 22q11DS and healthy controls. We examined neural responses to increasing intensities of two primary emotions—fear and disgust. Our main findings were that a) fusiform-extrastriate cortices and other components of ‘face responsive’ networks are engaged by fearful, disgusted, and neutral expressions both in young individuals with 22q11DS and in healthy controls; b) children with 22q11DS, like healthy controls, show increased activity in visual cortices and other brain regions with increasing intensity of fearful expressions; c) but, compared to controls, children with 22q11DS display a different pattern of modulation (i.e. increased activation) of right extrastriate cortical activity with increasing intensity of disgusted expressions.; and d) despite these between-group differences in the modulation of brain activity by emotion type and intensity, there were no brain regions where young people with 22q11DS showed greater activation to facial expressions of any type or intensity compared to typically developing controls. This is in line with previous findings of reduced FG response to faces in young people with 22q11DS [78] and is, in part, consistent with prior findings of frontal hyporesponsiveness to mixed facial expressions of emotion in adults with 22q11DS [75]. However, while the latter study reported higher activation of posterior regions in adults with 22q11DS compared to controls [75], as noted, we found no brain regions where the neural activations were higher in the 22q11DS group.

In addition, between-group differences in regional brain activation to facial expressions appear to be directly related to difficulties in social behaviour. For example, we found a negative correlation between brain activity and SDQ total difficulties score (a measure of difficulties in behaviour, emotions, and relationships) in the left precentral gyrus (BA 4) (for fear) and in the left FG (BA 19), right lingual gyrus (BA 18), and bilateral cerebellum (for disgust). Consequently, reduced responsiveness of these ‘face responsive’ regions to facial expressions of fear and disgust may contribute to the difficulties in social behaviour in people with 22q11DS. However, given the small sample size in the current study, further studies with larger samples will be required to validate this finding.

Similar findings of reduced activations of fusiform-extrastriate cortices to emotional and neutral facial expressions have been reported in an event-related fMRI study of people with ASD compared to healthy controls, using a similar incidental facial emotion processing task [103]. It has been suggested that fusiform-extrastriate hypoactivation in people with ASD may result from a failure of feedback modulation from limbic structures [63]. Similarly, fusiform-extrastriate hypoactivation in young people with 22q11DS may also relate to amygdala and other limbic abnormalities. For example, there is evidence of a lack of amygdala modulation by fearful expressions in young people with 22q11DS [78], while anomalies in limbic structures, along with other regions, have been reported from studies in 22q11DS [87, 104, 105]. The non-uniform pattern of between-group differences across emotion types and intensities in our study suggests that reduced feedback modulation from the amygdala may not be the sole explanation for our findings in 22q11DS. An alternative possibility is that limbic modulation of visual cortices involves the amygdala acting in concert with other limbic structures depending on the type and intensity of emotion. This hypothesis would be consistent both with the view that the amygdala is involved in conferring salience on a wide range of social and non-social stimuli [106] and with studies that reported differential activation of limbic structures in response to distinct facial emotion types—such as the amygdala for fear [107] and insula for disgust [108]. Hence, the between-group differences we found between people with 22q11DS and controls in visual cortical activation may reflect differences in modulatory input from limbic and other brain regions that vary with emotion type and intensity.

Compared to controls, the 22q11DS group also showed reduced activation in the anterior cingulate gyrus (BA 32/24). The dorsal region of the anterior cingulate cortex (BA 24/32) has been implicated in executive function, such as selective attention and planning, as well as in the regulation of affective states [46]. Hence, hypoactivation of the anterior cingulate gyrus in response to a range of expression types and intensities may relate to difficulties in regulating affective responses to emotional stimuli and in integrating affective responses to expressions with the executive control of behaviour.

Reduced brain activation in the 22q11DS group was also found in the superomedial prefrontal cortex (BA 6). Activation of this area in response to facial emotion stimuli has been reported in neuroimaging studies employing similar facial emotion processing tasks [60, 103]. In addition, BA 6 is also active during the production of empathetic facial expressions [109–111]. Relative hypoactivation of superomedial prefrontal regions in 22q11DS may therefore be related both to the failure to recognize and/or affectively respond to the facial displays of others and to the lack of facial expression reported in people with 22q11DS [17, 30].

Activations in the cerebellum during facial emotion and other affective processing tasks have been reported by prior human studies [57, 64, 65, 67, 103, 112–114]. While no consistent differential brain activations in facial emotion processing tasks have been found [57], it has been suggested that the cerebellum may contribute both to empathic processing [115, 116] and to the generation of affective states [104, 117]. Thus, the lower cerebellar activation observed in children with 22q11DS as compared to the healthy controls may result in diminished affective responsiveness to facial displays of expression. Cerebellar hypoactivation to facial expressions may also be associated with problems in social behaviour. For example, we found a significant negative correlation between adaptive social behaviour (as measured by the SDQ total difficulty scores which measure difficulties in behaviour, emotions, and peer relationships) and the left cerebellar activation to disgust in people with 22q11DS [103].

One possible explanation for differences in modulation of social brain regions by different types and intensity of facial expression may be variation in dopamine metabolism—for example, associated with variation in catechol-O-methyl transferase (COMT), a methylation enzyme that metabolizes catecholamines (including dopamine) [118]. In other words, dopamine levels in social brain regions could be abnormal in people with 22q11DS who have haploinsufficiency in COMT [119–122]. We will address this issue in future larger studies.

Alternatively, differences in function of social brain regions may be explained by differences in brain anatomy. We have previously reported a relationship between differences in regional brain volume and social behaviour in young people with 22q11DS [87]. Thus, larger studies are required to investigate the relationship between differences in anatomy and function (if any).

There are several limitations in our study. For example, the sample size was relatively small, and the controls scored significantly higher on tests of intellectual functioning compared to people with 22q11DS (FSIQ: 114 vs. 66). Hence, it could be suggested that our results may be explained by differences in intellectual ability, and it might therefore have been better to have included an IQ-matched non-deleted control group who were also matched on social and environmental factors. However, we compared people with 22q11DS to healthy controls (including siblings of the 22q11DS participants, when available) because we wanted to find out how brain activation in people with 22q11DS differs from those with optimal brain development. Also, both groups showed activations in brain regions reported by others to be specifically implicated in facial emotion processing [59, 60], and the pattern of the results remained unchanged even when we analysed the group differences using FSIQ as a covariate. Furthermore, unrelated controls with intellectual disabilities would most likely have comprised a heterogeneous population with a large variety of genetic and environmental/social differences. Hence, where possible, we used sibling controls to account, as best we could, for environmental and social differences. Thus, the significantly higher activations in the healthy control group probably cannot be solely attributed to environmental and/or socio-economic factors. Another point regarding the use of siblings as controls is that they may not represent typically developing healthy children. For example, imaging studies on individuals with autism have shown that non-autistic siblings of individuals with autism showed patterns of brain activation that are similar to those with autism [123] or different from those with autism and non-sibling controls [124]. Future research could therefore include sibling and non-sibling controls to address the issue of whether there are any differences in brain activation between sibling and non-sibling controls in the case of 22q11DS.

We did not acquire behavioural data about accuracy of facial emotion recognition because we employed incidental (gender discrimination) rather than explicit (emotion recognition) task. An incidental emotion processing task was chosen because emotion appraisal in routine social interaction often occurs automatically (without conscious deliberation) [125]. Therefore, we believe our paradigm to be more appropriate for investigating between-group differences in brain systems that are routinely engaged in social interaction.

We found widespread limbic and subcortical activations in our face versus fixation cross contrasts for both disgusted and fearful emotion across different levels of intensity (see Additional file 1) and between-group differences in activation of the insula to disgusted expressions (see the “Results” section). In contrast, we failed to find activation of the amygdala in any of our contrasts. However, not all previous studies of facial fear perception in healthy participants have demonstrated amygdala activation [126]. Similarly, the previous study on facial emotion processing in adults with 22q11DS reported higher insula activation in the control group, but they found no between-group difference in amygdala activations [75]. These results may not necessarily reflect absence of amygdala activation but, rather, limitations of MRI acquisition and the relatively small sample size in these studies. However, despite the relatively small sample size (*n* = 14), our results demonstrate that we had sufficient statistical power to detect differences in activation. Also, the areas we report are likely to remain differentially active even if additional active areas are revealed by increased sample size and optimized MRI acquisition parameters. Moreover, the results we report are likely to represent true activations because of the use of a conservative analysis and thresholding method to reduce the risk of Type 1 errors (see the “Methods” section).

## Conclusions

Both children and adolescents with 22q11DS and healthy controls demonstrated activation in ‘face responsive’ regions, including fusiform-extrastriate cortices, anterior cingulate cortex (BA 24), and superomedial prefrontal cortex (BA 6) in response to facial expressions of emotion (fear and disgust). However, the patterns of these activations vary depending on emotion type and intensity and differ between groups. Furthermore, compared to healthy controls, children with 22q11DS consistently showed significantly lower brain activations for both emotion types, across intensities, in these regions. Activity in some of these regions (e.g. left precentral gyrus (BA 4) to fear, and left FG (BA 19) and right lingual gyrus (BA 18) to disgust) also negatively correlated with the extent of social difficulties. Hypoactivation in these regions may therefore partly explain the social impairments of children with 22q11DS. However, further studies are required to determine the relationship between these findings and the social impairments of people with 22q11DS and how these abnormalities arise and change with age.

## Authors’ information

Rayna Azuma and Quinton Deeley are joint first authors.

## Electronic supplementary material

Additional file 1: Table 6: Contrasts of neural responses to neutral faces versus fixation cross; **Table 7.** Contrasts of neural responses to intense fearful face versus fixation cross; **Table 8.** Contrasts of neural responses to intense disgusted face versus fixation cross. (DOC 38 KB)

## Abbreviations

22q11DS: 22q11.2 deletion syndrome
ADD: attention deficit disorder
ADHD: attention deficit hyperactivity disorder
ANOVA: analysis of variance
ASD: autistic spectrum disorder
ASQ: Autism Screening Questionnaire
BA: Brodmann area
COMT: catechol-O-methyl transferase
EPI: echoplanar imaging
FG: fusiform gyrus
fMRI: functional magnetic resonance imaging
FSIQ: Full Scale IQ
ISI: interstimulus interval
SDQ: Strengths and Difficulties Questionnaire
VCFS: velo-cardio-facial syndrome
WISC-III: Wechsler Intelligence Scale for Children-III.
