# Supplementary material for: An fMRI study of facial emotion processing in children and adolescents with 22q11.2 deletion syndrome
Source: J Neurodev Disord. 2015 Jan 2;7(1):1. doi: 10.1186/1866-1955-7-1 (PMC4429366; doi:10.1186/1866-1955-7-1)
Supplement: Supplementary file 1 — Additional file 1: Table 6: Contrasts of neural responses to neutral faces versus fixation cross; Table 7. Contrasts of neural responses to intense fearful face versus fixation cross; Table 8. Contrasts of neural responses to intense disgusted face versus fixation cross. (DOC 38 KB) [file 11689_2014_109_MOESM1_ESM.doc]

# Additional File 1

**Table 6. Contrasts of neural responses to neutral faces versus fixation cross**

**Size Tal(x) Tal(y) Tal(z) BA Cerebral Region**

**22q11DS Group**

75 22 -63 -24 Cerebellum

68 25 -81 -13 19 Fusiform Gyrus

50 -36 -30 37 40 Inferior Parietal Lobule

48 -32 -30 53 3 Postcentral Gyrus

37 -32 -30 59 4 Precentral Gyrus

35 14 -67 4 18 Lingual Gyrus

33 36 -33 42 40 Inferior Parietal Lobule

23 40 -78 9 19 Middle Occipital Gyrus

18 29 -33 26 13 Insula

12 54 -11 20 43 Postcentral Gyrus

11 25 -67 15 18 Cuneus

9 29 -67 20 31 Posterior Cingulate

9 51 -15 31 4 Precentral Gyrus

8 -29 -52 26 39 Superior Temporal Gyrus

8 25 -70 37 7 Precuneus

7 32 -78 -2 18 Inferior Occipital Gyrus

6 54 -11 9 22 Superior Temporal Gyrus

5 43 -67 -2 37 Inferior Temporal Gyrus

**Normal Controls**

65 -36 -70 -24 Cerebellum

56 32 -63 -24 Cerebellum

54 -32 -30 48 3 Postcentral Gyrus

41 -4 15 37 32 Cingulate Gyrus

38 -43 0 -2 13 Insula

38 32 -78 -13 19 Fusiform Gyrus

35 4 -7 48 6 Medial Frontal Gyrus

29 -29 -30 53 4 Precentral Gyrus

28 40 7 20 13 Insula

25 25 -85 -7 18 Middle Occipital Gyrus

23 -22 0 -7 Putamen

22 4 15 42 32 Cingulate Gyrus

21 7 -22 4 Thalamus

20 -25 -81 -7 18 Middle Occipital Gyrus

18 -4 -26 -13 Brainstem

17 -7 -15 15 Thalamus

16 22 15 -7 Putamen

16 18 11 -13 47 Inferior Frontal Gyrus

12 4 -70 26 31 Precuneus

12 -40 11 26 9 Middle Frontal Gyrus

11 -14 4 -13 34 Subcallosal Gyrus

11 40 -22 48 3 Postcentral Gyrus

11 -54 -26 31 40 Inferior Parietal Lobule

9 -22 -26 -7 28 Parahippocampal Gyrus

9 -32 19 -7 47 Inferior Frontal Gyrus

6 4 22 20 24 Anterior Cingulate

6 36 41 15 10 Middle Frontal Gyrus

Size = number of voxels comprising cluster

Largest active area for a given brain structure reported, derived from decomposition of each 3D cluster into contiguous slices, 5.72 mm diameter in the z dimension. Statistical thresholds adjusted so as to get less than one false positive cluster per map.

**Table 7. Contrasts of neural responses to intense fearful face versus fixation cross**

**Size Tal(x) Tal(y) Tal(z) BA Cerebral Region**

**22q11DS Group**

69 36 -59 -18 Cerebellum

63 -36 -33 42 40 Inferior Parietal Lobule

60 -36 -33 53 40 Postcentral Gyrus

35 -36 -67 -13 19 Fusiform Gyrus

30 29 -78 -7 18 Middle Occipital Gyrus

27 4 7 42 32 Cingulate Gyrus

20 7 -22 48 6 Medial Frontal Gyrus

19 -18 -85 -7 18 Lingual Gyrus

17 -36 -44 -29 Cerebellum

16 25 -78 9 17 Cuneus

11 32 -78 -2 18 Inferior Occipital Gyrus

9 -47 -4 37 6 Precentral Gyrus

6 4 -30 20 23 Posterior Cingulate

6 11 30 20 32 Anterior Cingulate

6 -25 -85 4 18 Middle Occipital Gyrus

6 29 -74 15 31 Precuneus

**Normal Controls**

75 -43 -30 37 40 Inferior Parietal Lobule

70 -36 -30 48 3 Postcentral Gyrus

63 -43 7 4 13 Insula

62 25 -81 -18 Cerebellum

62 -36 -74 -18 Cerebellum

53 7 11 48 6 Superior Frontal Gyrus

50 40 15 20 46 Middle Frontal Gyrus

48 4 15 37 32 Cingulate Gyrus

48 7 11 42 32 Medial Frontal Gyrus

47 -18 11 -2 Putamen

45 29 19 4 Claustrum

40 40 15 9 13 Insula

38 18 -85 -13 18 Lingual Gyrus

36 -51 4 9 44 Precentral Gyrus

34 4 -4 -2 Hypothalamus

31 47 19 15 45 Inferior Frontal Gyrus

27 40 -26 48 3 Postcentral Gyrus

26 40 0 26 6 Precentral Gyrus

22 22 -85 -7 18 Middle Occipital Gyrus

22 -43 -70 -7 19 Inferior Occipital Gyrus

21 18 -26 -7 28 Parahippocampal Gyrus

11 -47 4 20 9 Inferior Frontal Gyrus

10 43 -30 37 40 Inferior Parietal Lobule

10 43 -30 42 40 Inferior Parietal Lobule

9 51 15 -7 38 Superior Temporal Gyrus

8 29 -74 9 30 Cuneus

8 -4 -26 -13 Brainstem

8 -4 -15 31 23 Cingulate Gyrus

6 29 -70 20 31 Precuneus

5 40 -48 -13 37 Fusiform Gyrus

5 22 4 -13 34 Subcallosal Gyrus

Size = number of voxels comprising cluster

Largest active area for a given brain structure reported, derived from decomposition of each 3D cluster into contiguous slices, 5.72 mm diameter in the z dimension. Statistical thresholds adjusted so as to get less than one false positive cluster per map.

**Table 8. Contrasts of neural responses to intense disgusted face versus fixation cross**

**Size Tal(x) Tal(y) Tal(z) BA Cerebral Region**

**22q11DS Group**

89 25 -85 -7 18 Middle Occipital Gyrus

57 32 -52 -24 * Cerebellum

56 -29 -33 53 3 Postcentral Gyrus

53 -4 -30 -2 Thalamus

53 7 -15 4 Thalamus

52 0 0 48 6 Medial Frontal Gyrus

48 -32 -33 42 40 Inferior Parietal Lobule

47 7 -26 -13 Brainstem

41 -40 -74 -13 19 Fusiform Gyrus

40 29 -74 -13 19 Fusiform Gyrus

39 7 19 37 32 Cingulate Gyrus

39 0 0 42 24 Cingulate Gyrus

30 -32 -44 -24 Cerebellum

16 43 -67 4 37 Middle Occipital Gyrus

16 25 -81 9 17 Cuneus

15 -40 -78 -7 18 Middle Occipital Gyrus

9 25 -81 15 19 Middle Occipital Gyrus

6 -22 -78 15 17 Cuneus

5 -22 -63 -2 19 Lingual Gyrus

5 32 -70 15 31 Precuneus

**Normal Controls**

76 32 -63 -18 Cerebellum

73 -36 -70 -18 Cerebellum

53 0 -4 48 6 Medial Frontal Gyrus

45 54 4 4 22 Superior Temporal Gyrus

44 -29 -33 48 3 Postcentral Gyrus

43 -32 -78 -13 19 Fusiform Gyrus

40 54 4 9 44 Precentral Gyrus

37 -25 -59 31 7 Precuneus

35 22 -85 -13 18 Fusiform Gyrus

31 -32 -67 -35 Inferior Semi-Lunar Lobule

31 4 4 42 24 Cingulate Gyrus

24 0 -19 42 31 Paracentral Lobule

23 47 7 26 9 Inferior Frontal Gyrus

20 51 -37 4 22 Middle Temporal Gyrus

17 22 -85 -7 18 Middle Occipital Gyrus

17 -25 -85 -7 18 Middle Occipital Gyrus

16 40 11 15 13 Insula

15 54 -30 -13 20 Inferior Temporal Gyrus

13 29 -70 26 31 Precuneus

12 29 -56 31 39 Angular Gyrus

10 -11 15 31 24 Cingulate Gyrus

9 -54 -26 26 40 Inferior Parietal Lobule

8 -54 -19 15 41 Transverse Temporal Gyrus

6 -4 -19 53 6 Medial Frontal Gyrus

Size = number of voxels comprising cluster

Largest active area for a given brain structure reported, derived from decomposition of each 3D cluster into contiguous slices, 5.72 mm diameter in the z dimension. Statistical thresholds adjusted so as to get less than one false positive cluster per map
